# Carbohydrate-Free Peach (*Prunus persica*) and Plum (*Prunus domestica*) Juice Affects Fecal Microbial Ecology in an Obese Animal Model

**DOI:** 10.1371/journal.pone.0101723

**Published:** 2014-07-09

**Authors:** Giuliana D. Noratto, Jose F. Garcia-Mazcorro, Melissa Markel, Hercia S. Martino, Yasushi Minamoto, Jörg M. Steiner, David Byrne, Jan S. Suchodolski, Susanne U. Mertens-Talcott

**Affiliations:** 1 Department of Nutrition and Food Science, Texas A&M University, College Station, Texas, United States of America; 2 Facultad de Medicina Veterinaria y Zootecnia, Universidad Autónoma de Nuevo León, General Escobedo, Nuevo León, México; 3 Gastrointestinal Laboratory, Texas A&M University, College Station, Texas, United States of America; 4 Department of Horticultural Sciences, Texas A&M University, College Station, Texas, United States of America; 5 Veterinary Physiology and Pharmacology, Texas A&M University, College Station, Texas, United States of America; University of Quebect at Trois-Rivieres, Canada

## Abstract

**Background:**

Growing evidence shows the potential of nutritional interventions to treat obesity but most investigations have utilized non-digestible carbohydrates only. Peach and plum contain high amounts of polyphenols, compounds with demonstrated anti-obesity effects. The underlying process of successfully treating obesity using polyphenols may involve an alteration of the intestinal microbiota. However, this phenomenon is not well understood.

**Methodology/Principal Findings:**

Obese Zucker rats were assigned to three groups (peach, plum, and control, n = 10 each), wild-type group was named lean (n = 10). Carbohydrates in the fruit juices were eliminated using enzymatic hydrolysis. Fecal samples were obtained after 11 weeks of fruit or control juice administration. Real-time PCR and 454-pyrosequencing were used to evaluate changes in fecal microbiota. Over 1,500 different Operational Taxonomic Units at 97% similarity were detected in all rats. Several bacterial groups (e.g. *Lactobacillus* and members of Ruminococcacea) were found to be more abundant in the peach but especially in the plum group (plum juice contained 3 times more total polyphenolics compared to peach juice). Principal coordinate analysis based on Unifrac-based unweighted distance matrices revealed a distinct separation between the microbiota of control and treatment groups. These changes in fecal microbiota occurred simultaneously with differences in fecal short-chain acids concentrations between the control and treatment groups as well as a significant decrease in body weight in the plum group.

**Conclusions:**

This study suggests that consumption of carbohydrate-free peach and plum juice has the potential to modify fecal microbial ecology in an obese animal model. The separate contribution of polyphenols and non-polyphenols compounds (vitamins and minerals) to the observed changes is unknown.

## Introduction

Obesity is a critical health issue worldwide affecting both industrialized and developing nations. Several factors have been associated with the increasing prevalence of obesity, including diminished physical exercise and an increased consumption of saturated fats and refined carbohydrates. Obesity is associated with multiple clinical complications and diseases including insulin resistance, hypertension, inflammation, oxidative stress, and dyslipidemia [1]–[4].

Polyphenols are a diverse group of compounds that are ubiquitous in the plant kingdom [5]. Over the last few years, the beneficial effects associated with the consumption of polyphenols have been widely studied [6]–[9]. Several *in vitro* and *in vivo* studies have demonstrated the anti-oxidant and anti-inflammatory activities of polyphenolics [10]–[12], some of which have also been shown to possess anti-lipidemic and anti-obesity effects, including suppression of adipogenesis and adipocyte proliferation, inhibition of fat absorption, as well as modulation of energy metabolism and inflammation [6], [13]. Interestingly, a growing number of investigations suggest that dietary polyphenols can modulate the composition and metabolic activity of intestinal microorganisms [14]–[21], which may be, at least in part, involved in the underlying mechanisms for the associated health benefits. This hypothesis is supported by the close association between energy harvest, obesity, and the complex assembly of microorganisms residing in the intestinal tract [22], [23].

Obesity has been linked to the composition of the gut microbiota but this relationship is not completely understood. Moreover, dietary interventions aiming to treat obesity have mostly focused on non-digestible carbohydrates [23]. Although the effect of polyphenols on the intestinal microbiota has been studied using culture and molecular techniques [15], [16], [24], [25], research is needed to determine whether these widely available compounds are capable of modulating the gut microbiota in obese individuals. Additionally, the gut microbiota consists of hundreds of microbial taxa, an ecosystem that can only be fully approached using high-throughput sequencing systems. Unfortunately, very few papers are available that have made use of these technologies to obtain a better insight on the effect of polyphenolics-rich fruits on the intestinal microbiota [26].

The use of animal models is common to study the gut microbiota because mammals (humans included) share the most predominant gut phylotypes and therefore the obtained results may help guide future interventions, either dietary or therapeutic, in human populations. Zucker rats possess a mutation in the leptin receptor and develop metabolic syndrome symptoms, including insulin resistance and dyslipidemia, at 4–5 weeks of age. This animal model has been very well characterized as a model of obesity and therefore makes it attractive for studies of the gut microbiota [27]. The present study aimed to investigate the effect of carbohydrate-free peach and plum juice on fecal microbial ecology using obese Zucker rats as the animal model. Animals were assigned to three groups (peach, plum and control obese), the lean wild-type was used as control lean. Quantitative real-time PCR revealed a significantly higher abundance of the phylum Bacteroidetes, the family Ruminococcacea, and the genera *Faecalibacterium*, *Lactobacillus*, and *Turicibacter* in the plum group (3 times more polyphenolics than peach) when compared to the control and the lean groups. These changes were accompanied by a significant difference between control and treatment groups in principal coordinate analysis (based on Unifrac-based unweighted distance matrices), differences in fecal fatty acids among the animal groups as well as by a significantly lower body weight in the plum group.

## Material and Methods

### Ethics statement

Experiments were approved by the Institutional Animal Care and Use Committee at Texas A&M University (AUP#2010-138). This research complies with the ‘Animal Research: Reporting of *In Vivo* Experiments’ (ARRIVE) guidelines (Checklist S1) [28].

### Study design

Male Zucker-Lepr^fa^/Lepr^+^ heterozygotes rats were used to evaluate the effects of peach and plum juice on the obese fecal microbiota. The lean Zucker-Lepr+ (Wild Type) rats were used as negative controls. Animals were purchased from Harlan Laboratories (Houston, TX) at 5–6 weeks age and maintained in a ventilated rack system with food and water provided *ad libitum*. All obese Zucker rats were the same age and arrived at the same time in our laboratory. After an acclimation period of seven days, the obese Zucker rats were allocated to three groups (n = 10 each) namely control, peach, and plum. The wild type Zucker rat group (n = 10) was named lean. The control and lean groups received a control beverage containing water with glucose in the same concentration as the average concentration of reducing sugars in peach and plum juices (2.4%±0.1). Additionally, pH was adjusted to match the pH of juices using citric acid. Animals were housed in pairs (2 rats per cage) at 22–25°C under a 12 hours light cycle. All rats were visually inspected every day and body weight was recorded from all animals once a week.

### Preparation of peach and plum juices

The commercial varieties “Angeleno” plum and “Crimson Lady” peach were collected at a mature, firm stage of development from commercial packing houses near Fresno, CA and shipped next day to the Department of Horticultural Sciences, Texas A&M University, College Station, TX. Fruits were stored at 4°C on the day of arrival whereby the stone was removed and the edible flesh stored at −80 °C until juice preparation. Peach and plum juices were prepared by enzymatic hydrolysis of pureed pulp obtained with a food processor. In brief, fruit puree was heated up to 90°C to inactivate polyphenoloxidase enzymes, cooled down to 50–55°C and subjected to enzymatic hydrolysis for 2 h with a mixture of food-grade enzymes multicellulase complex and hemicellulases (ValidaseTRL), pectin esterase, depolymerase, cellulases, hemicellulases, and arabinase (Crystalzyme 200XL) kindly supplied by Valley Research (South Bend, IN). After enzymatic hydrolysis, clarified peach and plum juices were obtained by centrifugation at 5000 rpm for 5 min.

### Reducing sugars and total polyphenols

Reducing sugars were determined using dinitrosalicilic acid as a reagent against a standard curve of glucose [29]. Peach and plum juices contained 2.3±0.3% and 2.5±0.4% of reducing sugars respectively. Total polyphenols were quantified with Folin-Ciocalteu reagent (Fisher Scientific, Pittsburgh, PA) against a standard curve of gallic acid and expressed as mg gallic acid equivalents (GAE)/L [30]. Peach and plum juices contained 430±6.3 and 1,270±12.6 mg GAE/mL respectively.

### Fecal collection and DNA extraction

Fresh fecal samples were obtained from all rats at the end of the study (11 weeks of consumption of sugary water or peach or plum juices) and stored at −80 C until analysis. Total DNA was extracted and purified from 100 mg of fecal sample using a bead-beating phenol-chloroform method as previously described [31].

### Quantitative real-time PCR (qPCR)

The primary experimental outcome was the abundance of fecal microbiota, as determined by qPCR and pyrosequencing. qPCR analyses were performed to first investigate changes in specific bacterial groups among the animal groups. Briefly, PCR reaction mixtures (total of 10 µL) contained 5 µL of SsoFast EvaGreen supermix (Biorad Laboratories), 2.6 µL of water, 0.4 µL of each primer (final concentration: 400 nM), and 2 µL of adjusted (5 ng/μL) DNA. PCR conditions were 95°C for 2 min and 40 cycles at 95°C for 5 s and 10 s at the optimized annealing temperature (Table 1). A melt curve analysis was performed to verify the specificity of the primers using the following conditions: 1 min at 95°C, 1 min at 55°C, and 80 cycles of 0.5°C increments for 10 s each. Raw PCR data was normalized to the qPCR data for the total bacteria (universal primers F341 and R518) and all samples were run in duplicate as performed elsewhere [33].

**Table 1:** Oligonucleotides used in this study for qPCR analysis.

qPCR primers	Sequence (5′–3′)	Target	Annealing (°C)	Reference
UniF	CCTACGGGAGGCAGCAG	All bacteria	59	[32]
UniR	ATTACCGCGGCTGCTGG			
RumiF	ACTGAGAGGTTGAACGGCCA	Family Ruminococcaceae	59	[33]
RumiR	CCTTTACACCCAGTAAWTCCGGA			
FaecaliF	GAAGGCGGCCTACTGGGCAC	*Faecalibacterium*	60	[33]
FaecaliR	GTGCAGGCGAGTTGCAGCCT			
LacF	AGCAGTAGGGAATCTTCCA	*Lactobacillus*	58	[34]
LacR	CACCGCTACACATGGAG			
TuriciF	CAGACGGGGACAACGATTGGA	*Turicibacter*	63	[35]
TuriciR	TACGCATCGTCGCCTTGGTA			
CFB555f	CCGGAWTYATTGGGTTTAAAGGG	Bacteroidetes	60	[36]
CFB968r	GGTAAGGTTCCTCGCGTA			
BifF	TCGCGTCYGGTGTGAAAG	*Bifidobacterium*	60	[34]
BifR	CCACATCCAGCRTCCAC			

### 454-pyrosequencing

Bacterial tag-encoded FLX-titanium amplicon pyrosequencing (bTEFAP) was performed using the primers 28F (GAGTTTGATCNTGGCTCAG, forward) and 519R (GTNTTACNGCGGCKGCTG, reverse) targeting a semi-conserved region of the 16S rRNA gene at the Research and Testing Laboratory (Lubbock, TX). The Quantitative Insights in Microbial Ecology (QIIME) software platform (version 1.5.0) was used for processing and analysis of the sequences [37]. The process included chimera removal and denoising using UCHIME [38] and USEARCH [39], respectively, as well as removal of sequences that had low quality tags, primers, or ends, and failed to be at least 250 bp in length. The operational taxonomic units (OTUs) were defined as sequences with at least 97% similarity using the RDP classifier [40] in QIIME. Alpha and beta diversity measures were calculated using an equal number of sequences (2489, lowest number of sequences in a sample after removal of chimeric sequences) also using QIIME. Collection and sequence information has been submitted to the Sequence Read Archive (SRP029310).

### Fecal fatty acids analysis

Short-chain fatty acids (SCFA) and branched-chain fatty acids (BCFA) were measured in fecal samples in order to obtain a better understanding of the effect of peach and plum juice on the metabolic activity of the intestinal microbiota. Concentrations of SCFA (acetate, propionate, butyrate), and BCFA (isobutyrate, isovalerate, valerate) in feces were measured using a stable isotope dilution gas chromatography-mass spectrometry (GC-MS) assay as previously described [41], with some modifications. Briefly, the fecal samples were weighed and diluted 1∶5 in extraction solution (2N hydrochloric acid). After homogenization for 30 min at room temperature, fecal suspensions were centrifuged for 20 min at 2,100 g at 4°C. Supernatants were then collected using serum filters (Fisher Scientific Inc., Pittsburgh, Pa). Of each sample, 500 µl of supernatant were mixed with 10 µl of internal standard (200 mM heptadeuterated butyric acid) and extracted using a C18 solid phase extraction column (Sep-Pak C18 1 cc Vac Cartridge, Waters Corporation, Milford, MA). Samples were derivatized using *N*-tert-Butyldimethylsilyl-*N*-methyltrifluoroacetamide (MTBSTFA) at room temperature for 60 minutes. A gas chromatograph (Agilent 6890N, Agilent Technologies Inc, Santa Clara, CA) coupled with a mass spectrometer (Agilent 5975C, Agilent Technologies Inc, Santa Clara, CA) was used for chromatographic separation and quantification of the derivatized samples. Separation was achieved using a DB-1ms capillary column (Agilent Technologies Inc., Santa Clara, CA). The GC temperature program was as follows: 40°C held for 0.1 min, increased to 70°C at 5°C/min, 70°C held for 3.5 min, increased to 160°C at 20°C/min and finally increased to 280°C for 3 min at 35°C/min. The total run time was 20.5 min. The mass spectrometer was operated in electron impact positive-ion mode with selective ion monitoring at mass-to-charge ratios (*M/Z*) of 117 (acetate), 131 (propionate), 145 (butyrate and isobutyrate), 152 (deuterated butyrate; internal standard), and 159 (valerate and isovalerate). Quantification was based on the ratio of the area under the curve of the internal standard and each fatty acid. Results are reported as micromoles (μmol) per gram of wet feces.

### Statistical analysis

The experimental unit in this study was individual rats. Pyrosequencing data was used to determine any significant differences to the control using an analysis of similarities (ANOSIM) on the unweighted Unifrac distance matrix in PAST [42]. An unweighted Pair Group Method with Arithmetic Mean (UPGMA) hierarchical clustering was generated using QIIME to visualize clustering of samples. Differences in relative proportions of sequences (including the Firmicutes/Bacteroidetes ratio), alpha diversity indices, fecal fatty acids, body weight, and qPCR data were analyzed using an analysis of variance (ANOVA) or its non-parametric counterpart Kruskal-Wallis using JMP 9.0.0 (SAS Institute Inc.), depending on sample size, type of data, and/or normality of the residuals from the ANOVA. Multiple comparisons were adjusted by the Tukey-Kramer or the Dunn's method. A p<0.05 was considered for statistical significance. QIIME, JMP and R (version 2.15.2) were used to generate graphs.

## Results

Throughout the study (11 weeks) control and lean groups consumed an average of 50.6±8.7 and 46.0±7.9 mL water/animal-day, respectively, peach and plum groups consumed an average of 47.5±9.0 and 45.2±11.8 mL juice/animal-day respectively (Table S1). All rats remained clinically healthy during the study.

### qPCR analyses

**Figure 1:**
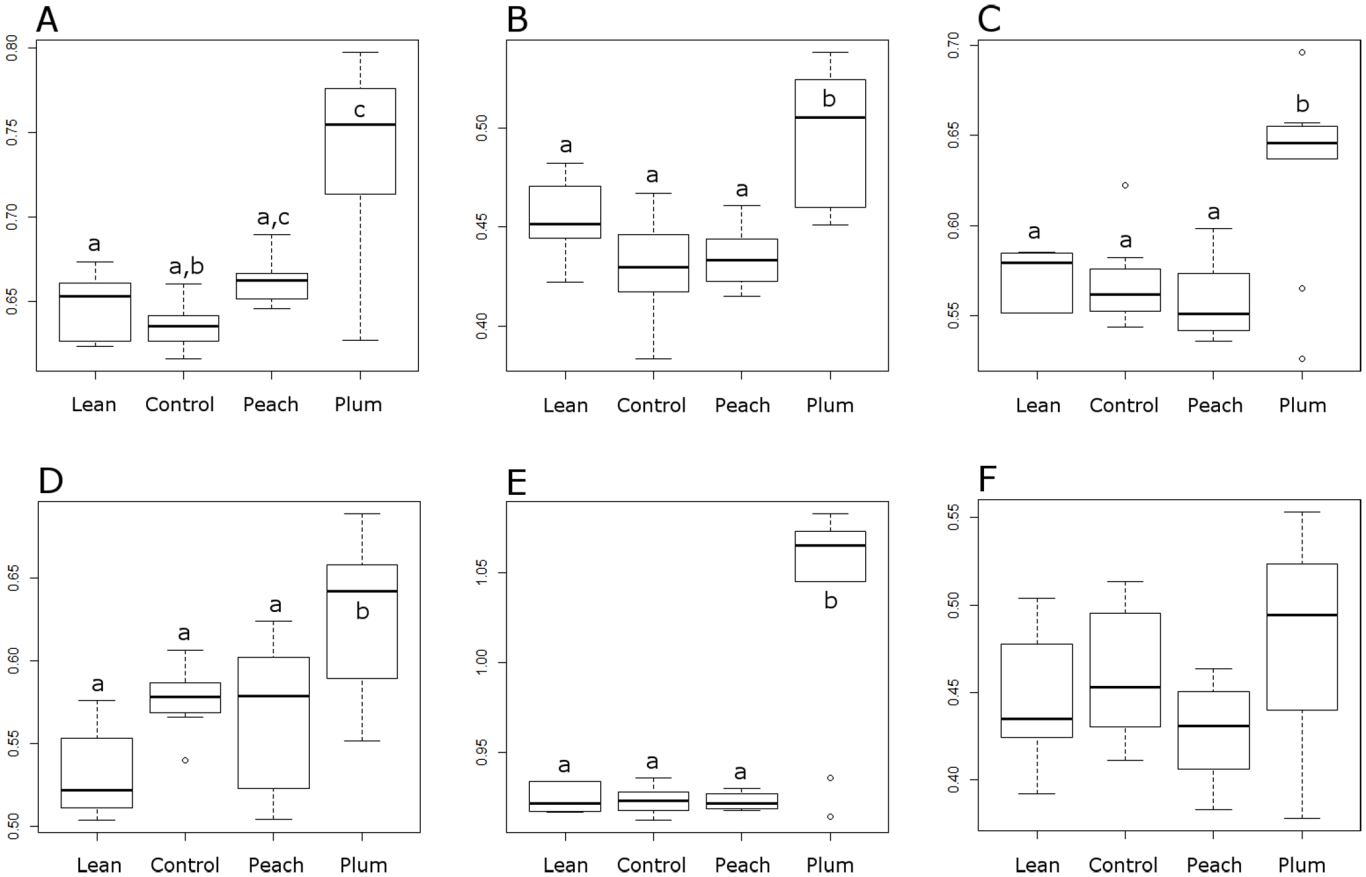
Quantitative real-time PCR results for Ruminococcaceae (family, A), *Faecalibacterium* (B), *Lactobacillus* (C), *Turicibacter* (D), Bacteroidetes (phylum, E) and *Bifidobacterium* (F) in the lean (n = 6), control obese (n = 8), peach (n = 7), and plum (n = 9) groups. Error bars represent the median and interquartile ranges (all results were normalized to qPCR data for total bacteria). Columns not sharing the same superscript are significantly different (p<0.05). *Significantly higher than all other groups.

qPCR analyses were performed on 6 samples from the lean group, 8 samples from the obese control group, 7 samples from the peach group, and 9 samples from the plum group. The reason for using a subset of samples obeyed availability of fecal DNA for all analysis. The abundance of Bacteroidetes (phylum) and the genera *Faecalibacterium*, *Lactobacillus*, and *Turicibacter* were found to be significantly higher in the plum group when compared to all other groups (p<0.05; Figure 1). The abundance of the family Ruminococcaceae was found to be significantly higher in the plum group when compared to both the control and the lean groups. Additionally, Ruminococcaceae was also significantly higher in the peach group when compared to the control group (Figure 1).

### bTEFAP

**Figure 2:**
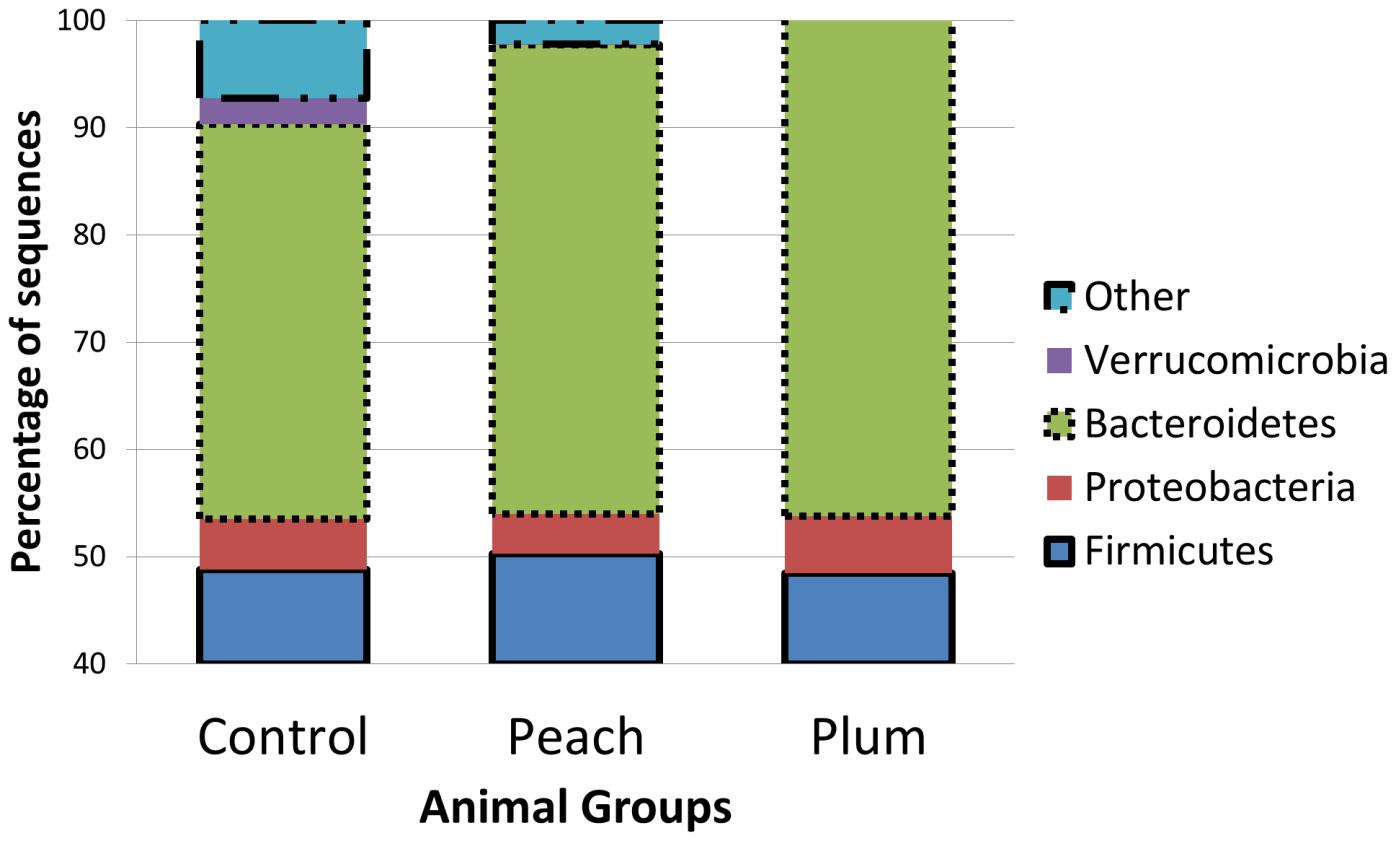
Composition of fecal microbiota in the control (n = 4), peach (n = 4) and plum (n = 4) groups at the phylum level. Bars represent median percentage of sequences. The y axis (percentage of sequences) was modified to also show the low abundant phyla.

Pyrosequencing was performed in an effort to investigate differences in the overall phylogenetic composition of the fecal microbiota among the animal groups. For this analysis, we analyzed 4 fecal DNA samples from the obese control group, 4 samples from the peach group and 4 samples from the plum group. Additionally, we also included one fecal DNA sample from a lean subject but the results from this separate analysis of all samples (control obese, peach, plum and the lean subject) are only provided as supporting information (Figures S1–S3, Table S2). A total of 60,798 non-chimeric good-quality 16S rRNA gene sequences were analyzed (average: 5,067 ±1,666 sequences per sample). The fecal microbiota of all rats was composed by 1,549 OTUs (97% similarity) from 12 distinctive bacterial phyla. Despite the high bacterial diversity, only four phyla (Firmicutes, Bacteroidetes, Verrucomicrobia, and Proteobacteria) accounted for more than 90% of all the obtained sequences (Figure 2). The Firmicutes/Bacteroidetes ratio was not significantly different among the control obese and treatment groups (p = 0.209, Figure 2).

**Figure 3:**
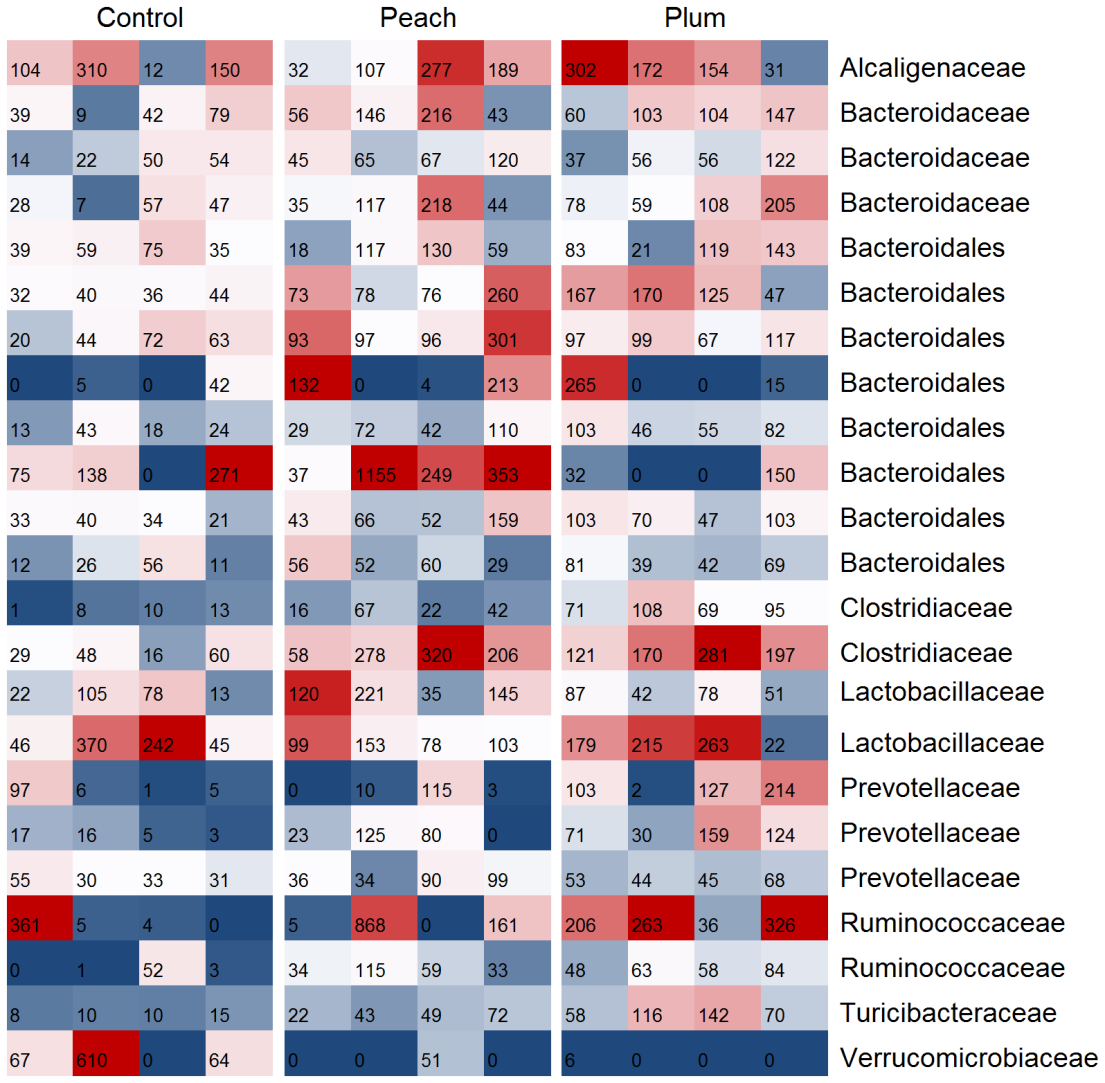
Heat map showing the most abundant operational taxonomic units (OTUs, at least 500 total) in the control (n = 4), peach (n = 4) and plum (n = 4) groups. Colors represent differences in relative abundance within samples (red: higher; white: median; blue: lower).

**Figure 4:**
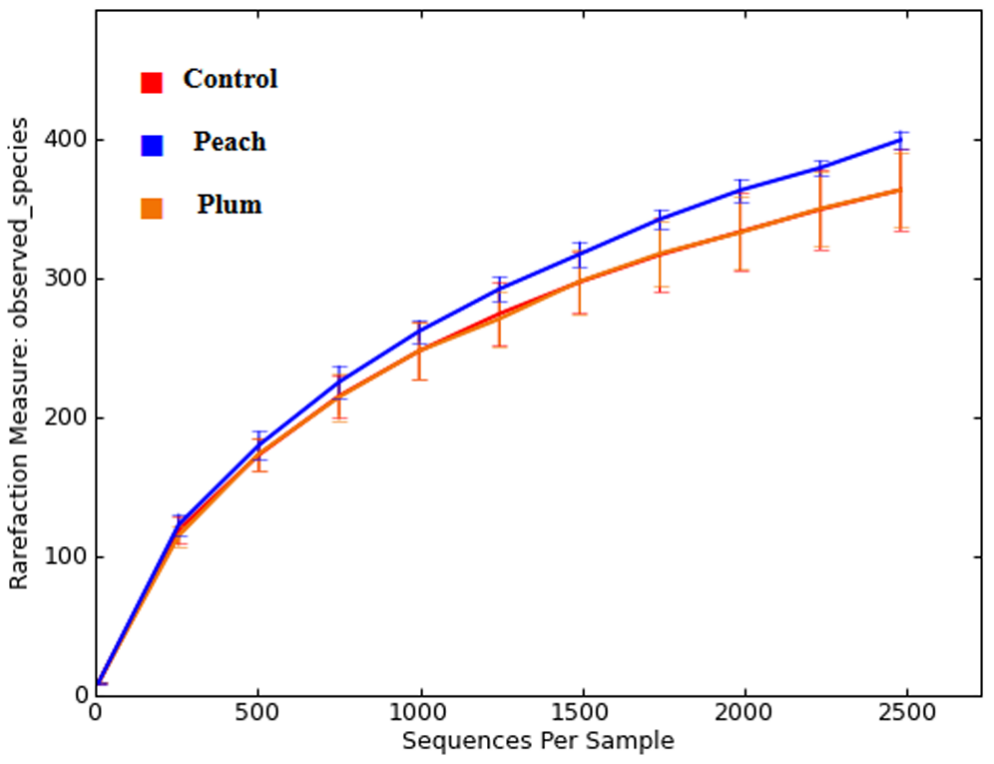
Rarefaction plots of 16S rRNA gene sequences obtained from fecal samples. Lines denote the average of each group; error bars represent the standard deviation. This analysis was carried out using a randomly selected 2489 sequences per sample.

A heat map of the most abundant OTUs (≥ 500 total in all samples analyzed) suggested differences in the relative abundance of various bacterial groups among the different animal groups (Figure 3) that confirmed the qPCR results (see above). Specifically, the relative abundance of OTUs from Turicibacteraceae was found to be high only in samples from the plum group. Moreover, most animals in the plum and the peach group had a high abundance of one unclassified Ruminococcaceae, and OTUs from several Bacteroidetes were also high only in the treatment groups (Figure 3). Despite these suggested dissimilarities in relative abundance of OTUs, there was no statistically significant difference in relative proportions of pyrosequencing reads (percentage of sequences) except for *Turicibacter* (Table S3), which was found to be significantly higher in both the peach and plum groups when compared to the control group. The genus *Akkermansia* (phylum Verrucomicrobia) was higher in the obese control group but this difference did not reach significance (p = 0.069, Table S3). Figure 4 illustrates the rarefaction curves for the control and the treatment groups. Alpha diversity indices were not significantly different among the animal groups (Table 2).

**Table 2:** Median (minimum-maximum) indices of bacterial diversity (Shannon Weaver and Chao1 3%) and richness (OTUs 3%) obtained from fecal samples of the control, peach and plum groups. *P* values come from the non-parametric Kruskal-Wallis.

	Control (n = 4)	Peach (n = 4)	Plum (n = 4)	p value
Chao1	500 (434–553)	600 (547–616)	485 (463–576)	0.0592
Shannon	6.9 (6.5–7.4)	7.1 (6.6–7.5)	7.0 (6.8–7.4)	0.8741
OTUs	359 (329–370)	401 (381–413)	355 (341–401)	0.1238

These estimates are based on 2489-sequences subsamples.

**Figure 5:**
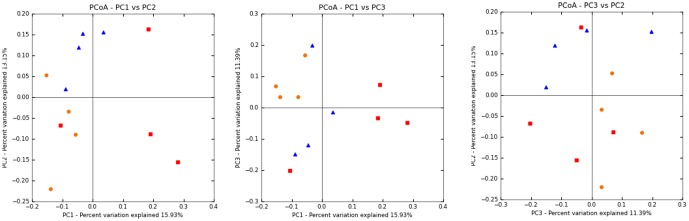
Principal Coordinate Analysis (PCoA) plots of the unweighted Unifrac distance matrix. The plots show each combination of the first three principal coordinates. Red (square): control; orange (circle): plum; blue (upright triangle): peach.

A Principal Coordinate Analysis (PCoA) analysis of the Unifrac-based unweighted distance matrices revealed useful information about the phylogenetic relationship among the fecal bacterial microbiota in the different animal groups (Figure 5). Most samples from the control obese group were separated from the peach and the plum samples in at least two of the combinations of coordinates (ANOSIM with 9999 permutations, p =  0.0012, Figure 5). It is known that when few independent factors are responsible for most of the variation, the first 2–3 coordinates explain most of the variation in the data [43]. In this study, the first three coordinates only described 41% of the variability, suggesting that many independent factors could have contributed to the observed variation in UniFrac distance values among the samples [43].

**Figure 6:**
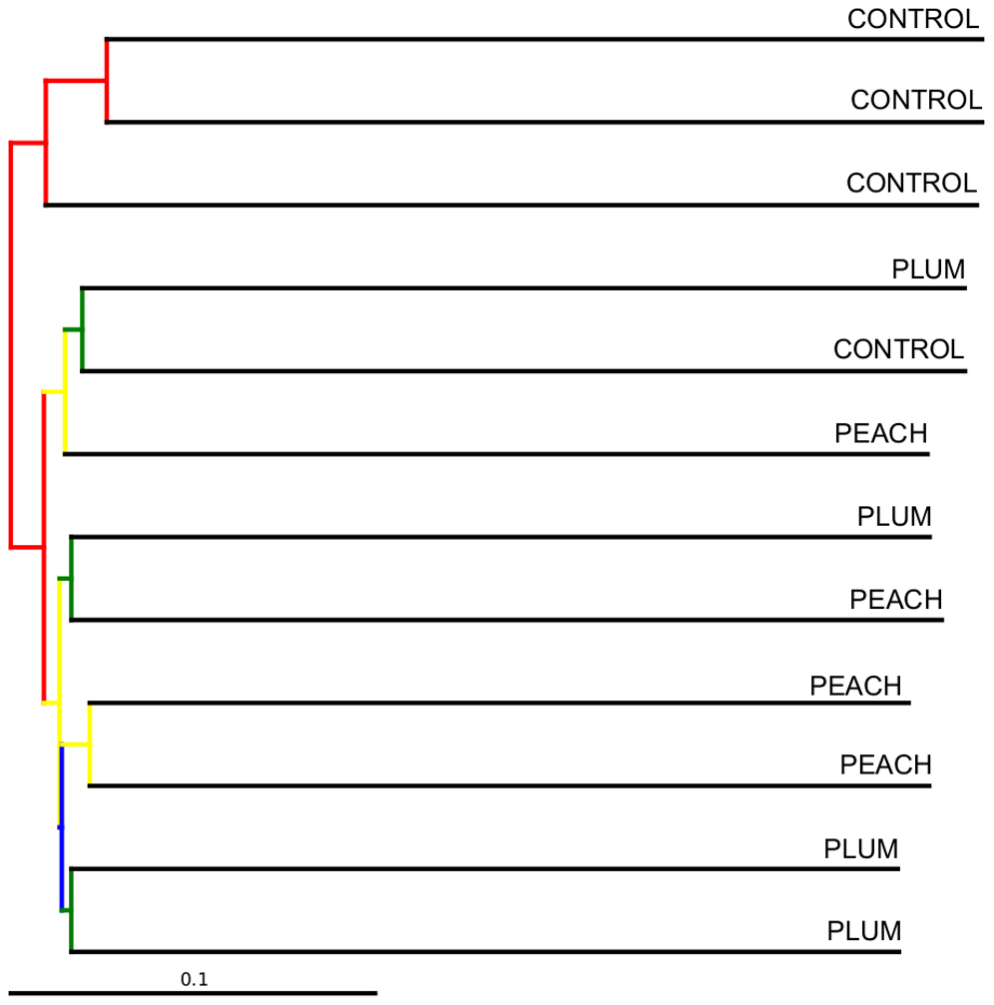
UPGMA hierarchical clustering using the unweighted Unifrac distance matrix. The colors represent different jackknife support: red (75–100% support); yellow (50–75%); green (25–50%); blue (<25% support). The bar represents community dissimilarity.

An UPGMA hierarchical clustering was created and suggested a distinctive clustering of all but one of the samples in the control group (75–100% jackknife support) (Figure 6). Expectedly, the sample from the control obese group that did not cluster with the rest of the control samples in the UPGMA hierarchical clustering was the same sample that remained independent in the PCoA analysis (Figure 5). There was not clear distinction (low jackknife support) among the samples from the peach and the plum groups (Figures 6), an observation that was also noted in the PCoA plots (Figure 5).

The analysis of all samples (control obese, treatment groups, and the one lean subject) revealed that the lean subject had higher indices of diversity and richness than any other sample analyzed (Table S2).

### Fecal fatty acids analysis

Fecal fatty acids were measured in a subset of samples from the peach (n = 6), plum (n = 8), control (n = 6) and lean (n = 5) groups. The samples from the obese control group had a significantly higher concentration of acetic and propionic acid when compared to the plum and the lean group (acetic acid) and the peach and the lean group (propionic acid) (p<0.05), respectively (Table S4). All other fecal fatty acids, including butyric acid, were not significantly different among the animal groups (Table S4).

### Body weight

Body weight at day 0 (beginning of experiment) was significantly different between the lean group and all other groups (data not shown). Animals in the plum group showed a significantly lower body weight (541.8 ±43.6) compared to control obese (644.4 ±39.3) and peach (611.1 ±39.4) group at week 11 (end of experiment, p<0.05, Table S1).

## Discussion

There has been an increased interest in the characteristics and potential modifications of the intestinal microbiota to improve health in obese individuals. However, little information is available investigating the effect of potentially beneficial nutrients on the obese microbiota. To our knowledge, this study is the first to report the effect of peach and plum juices on the intestinal microbiota of obese rats using molecular tools, including a high-throughput sequencing technique.

Obese individuals have been reported to harbor a distinctive intestinal microbiota when compared to non-obese subjects. For example, Ley *et al*. showed a lower proportion of Bacteroidetes and a higher proportion of Firmicutes in obese mice when compared with lean mice [44]. Likewise, it has been suggested that obesity is related to phylum-level changes in the microbiota and reduced bacterial diversity [45]. However, others have found either no difference in the proportions of the main phyla or a change in proportions that seemed to contradict the original observations by Ley *et al*. [23]. In this study, qPCR analyses revealed statistically significant differences in the abundance of several fecal bacterial groups between the treatment (peach and plum) groups compared to the control and lean groups, but there was no difference between the lean and the obese control groups. The reasons for this lack of difference between lean and obese subjects are unknown but other authors have proposed a role of inter-individual differences, methods of sample preparation or methods of bacterial analysis [46].

The study of intestinal microorganisms and their relationship with fat metabolism and obesity has received increased attention over the last few years. However, little is known about how to successfully manipulate the obese gut microbiota, previous studies mainly used non-digestible carbohydrates [23]. Using an obese animal model, this study suggest that the polyphenolics in the juices played a role in the observed changes because the plum juice contained 3 times more polyphenolics and the differences in fecal microbial ecology and body weight were more marked in the plum group. For example, we found a higher abundance of *Turicibacter* in the plum group and this bacterial group has received increased attention because of its close relationship with the immune system of the host [47]. Also, we found a higher abundance of Bacteroidetes in the plum group. As mentioned above, Ley *et al*. [44] and others have shown that lean individuals generally carry a higher abundance of this group. Interestingly, in the plum group we also found a higher abundance of *Faecalibacterium* and *Lactobacillus*, important and abundant members of the phylum Firmicutes [48]–[49]. Moreover, we found differences in the abundance of the genus *Akkermansia* (phylum Verrucomicrobia), whose abundance has been shown to decrease in obese and type 2 diabetic mice [50]. It is important to note that our results about *Akkermansia* are somehow in disagreement with previous studies where a high abundance of this bacterial group is associated with health [50]–[51]. In our study, the relative abundance of *Akkermansia* was higher (although not statistically, p = 0.069) in obese rats and the consumption of peach and plum extracts helped diminish its abundance (Table S3). This discrepancy may be explained by phenotypic differences among species within the genus or strains within the species as well as differences in the animal models utilized.

In order to obtain a better understanding of the effect of the peach and plum juices on the gut microbial ecosystem, we also measured SCFA and BCFA in fecal samples. Using an *in vitro* fecal culturing system, Bialonska *et al.* [18] showed that the inoculation of pomegranate polyphenols-rich extracts yielded significant increases in acetate, propionate and butyrate concentrations, as well as in the abundance of total bacteria, *Bifidobacterium* and *Lactobacillus* spp. Interestingly, the authors also inoculated the major pomegranate polyphenols (i.e., punicalagins) in the fecal cultures and did not observe changes in the abundance of fecal microorganisms and/or SCFA concentrations [18]. The authors of this study suggest that the effect of pomegranate extracts on fecal bacteria can be attributed to other non-punicalagins polyphenolics in pomegranate as well as glucose. Similarly, our data suggests that polyphenolics in the peach and plum juices have the potential to modify the composition of fecal SCFA concentrations *in vivo*. Moreover, the current study offers valuable information to the field of functional foods because carbohydrates were removed from the fruits. More detailed functional (metabolic) data, such as single-cell stable isotope probing, are necessary to research in more depth the complex bacterial interactions during the metabolism of polyphenolics inside the gut.

The cause of any difference in the fecal microbiota due to dietary polyphenols can be attributed to several factors. There is evidence suggesting that a proportion of dietary polyphenols can reach the large intestine in their original form [52]–[53], which are then subjected to microbial bioconversion [21]. Moreover, dietary polyphenols have the ability to inhibit the activity of pancreatic lipase, resulting in a reduced ability to absorb fat and consequently in a higher fecal fat content [54]–[56], and can promote fat oxidation and decrease lipogenesis [57]. Additionally, polyphenols are not considered as a primary energy source of microbial growth (compared to polysaccharides) [57] and possess both anti-microbial and growth-enhancing activities [15], [58]. Therefore, the differences observed in this study may have arisen from the bioconversion of polyphenols by the gut microbiota, modifications of the lipid metabolism, as well as anti-microbial and growth-enhancing effects. More research, using purified polyphenols and whole extracts from polyphenolics-rich foods, is needed to understand more in depth gut microbial metabolism of polyphenols.

This study analyzed the effect of carbohydrate-free peach and plum juices on the obese fecal microbiota. However, the juices most likely contained other compounds aside the polyphenolics, such as vitamins and minerals, as peach and plum are known to contain high concentrations of these nutrients. Although it is known that several members of the intestinal microbiota are capable of utilizing and synthesizing vitamins [59]–[60], very little is known about the effect of these and other specific nutrients on the gut microbiota. Nonetheless, we cannot rule out the possibility that vitamins, minerals and/or other compounds in the juices could have had a contribution on the changes we observed.

The relevance of the current study to human or veterinary medicine is debatable. There are similarities in the gut microbiota of different mammals based on gut type and diet [61]. Mice and rats also share many physiological similarities with humans and other mammals, and studies in these animal species can therefore be useful to human and veterinary medicine. However, it is difficult for this and other studies to generalize about the contribution of specific dietary nutrients to any change in the abundance or phylogenetic composition of the gut microbiota. For instance, in this study the prevention of weight gain could have been responsible for the changes in the microbiota instead or in addition to any change caused by direct microbial metabolism of the nutrients in the administered juices.

In summary, the current study suggests that the consumption of carbohydrate-free peach and plum juice has the potential to modify fecal bacterial composition in obese rats, as determined by qPCR and pyrosequencing. These changes occurred simultaneously with differences in fecal SCFA concentrations and a decrease in body weight in the plum group. Clinical research is needed to investigate the significance of our observations in preventing and treating human or veterinary patients with obesity.

## Supporting Information

Figure S1 **Principal Coordinate Analysis (PCoA) of the unweighted Unifrac distance matrix.** The plots show each combination of the first three principal coordinates. Red (square): control; green (circle): plum; orange (horizontal triangle): peach; blue (upright triangle): lean.
(PDF)

Figure S2 **UPGMA hierarchical clustering using the unweighted Unifrac distance matrix with the lean subject.** The colors represent different jackknife support: red (75–100% support); yellow (50–75%); green (25–50%); blue (<25% support). The bar represents community dissimilarity.
(PDF)

Figure S3 **Heat map showing the most abundant operational taxonomic units (OTUs, at least 500 total) in one lean subject, control, peach and plum groups.** Colors represent differences in relative abundance within samples (red: higher; white: median; blue: lower).
(PDF)

Table S1 **Total body weight at the end of the study (11 weeks of consumption of peach or plum juices), juice consumption and polyphenolics content in the control, peach, plum, and lean animal groups.**
(PDF)

Table S2 **Median (minimum-maximum) indices of bacterial diversity (Shannon Weaver and Chao1 3%) and richness (OTUs 3%) obtained from fecal samples of one lean subject, control, peach and plum groups.** P values come from the non-parametric Kruskal-Wallis.
(PDF)

Table S3 **Median (minimum-maximum) relative proportions of pyrosequencing tags (percentage of sequences) for the control, peach, and plum groups.** P values come from the non-parametric Kruskal Wallis test.
(PDF)

Table S4 **Median (minimum-maximum) concentrations (μmol/g of wet feces) of short-chain fatty acids obtained from fecal samples of the control, peach, plum, and lean groups.** P values come from the non-parametric Kruskal-Wallis.
(PDF)

Checklist S1 **ARRIVE checklist.**
(PDF)
