# Correction: Carbohydrate-Free Peach (*Prunus persica*) and Plum (*Prunus domestica*) Juice Affects Fecal Microbial Ecology in an Obese Animal Model

**DOI:** 10.1371/journal.pone.0106128

**Published:** 2014-08-18

**Authors:** The PLOS ONE Staff

The species name for the plum used in this study and listed in the title should be *Prunus salicina* and not *Prunus domestica*. The correct title is: Carbohydrate-Free Peach (*Prunus persica*) and Plum (*Prunus salicina*) Juice Affects Fecal Microbial Ecology in an Obese Animal Model.

The correct citation is: Noratto GD, Garcia-Mazcorro JF, Markel M, Martino HS, Minamoto Y, et al. (2014) Carbohydrate-Free Peach (*Prunus persica*) and Plum (*Prunus salicina*) Juice Affects Fecal Microbial Ecology in an Obese Animal Model. PLoS ONE 9(7): e101723. doi:10.1371/journal.pone.0101723
