# Supplementary material for: Carbohydrate-Free Peach (Prunus persica) and Plum (Prunus domestica) Juice Affects Fecal Microbial Ecology in an Obese Animal Model
Source: PLoS One. 2014 Jul 9;9(7):e101723. doi: 10.1371/journal.pone.0101723 (PMC4090149; doi:10.1371/journal.pone.0101723)
Supplement: Figure S3 — Heat map showing the most abundant operational taxonomic units (OTUs, at least 500 total) in one lean subject, control, peach and plum groups. Colors represent differences in relative abundance within samples (red: higher; white: median; blue: lower). (PDF) [file pone.0101723.s003.pdf]

Carbohydrate-free peach (*Prunus persica*) and plum (*Prunus domestica*) juice affects fecal microbial ecology in an obese animal model

Giuliana D. Noratto[1,a,b], Jose F. Garcia-Mazcorro[2,b], Melissa Markel[3], Hercia S. Martino[1], Yasushi Minamoto[3], Jörg M. Steiner[3], David Byrne[4], Jan S. Suchodolski[3] & Susanne U. Mertens-Talcott[1,5*]

1 Department of Nutrition and Food Science, Texas A&M University, College Station, Texas, United States of America

2 Facultad de Medicina Veterinaria y Zootecnia, Universidad Autónoma de Nuevo León, General Escobedo, Nuevo León, México

3 Gastrointestinal Laboratory, Texas A&M University, College Station, Texas, United States of America

4 Department of Horticultural Sciences, Texas A&M University, College Station, Texas, United States of America

5 Veterinary Physiology and Pharmacology, Texas A&M University, College Station, Texas, United States of America

[a] Current address: School of Food Science, Washington State University, USA.

[b] These authors contributed equally to this study.

*** Email**: SMTalcott@tamu.edu

Lean	Control				Peach				Plum				
31	1	2	6	7	11	15	16	40	26	27	29	41	
64	104	310	12	150	32	107	277	189	302	172	154	31	Alcaligenaceae
152	14	18	33	28	63	13	35	61	6	66	75	61	Bacteroidaceae
129	28	7	57	47	35	117	218	44	78	59	108	205	Bacteroidaceae
19	39	10	42	79	56	146	217	43	60	103	104	147	Bacteroidaceae
124	14	22	50	54	45	65	67	120	37	56	56	122	Bacteroidaceae
112	32	40	36	44	73	78	77	260	167	170	125	47	Bacteroidales
113	21	45	72	63	96	97	98	302	97	99	67	118	Bacteroidales
56	12	26	56	11	56	52	60	29	81	39	42	69	Bacteroidales
47	13	43	18	24	29	73	42	110	105	46	56	83	Bacteroidales
13	39	59	75	35	18	117	130	59	83	21	119	143	Bacteroidales
87	33	40	34	22	44	66	52	161	106	70	48	104	Bacteroidales
15	75	138	0	271	37	1155	249	353	32	0	0	150	Bacteroidales
42	1	8	10	13	16	67	22	42	71	108	69	95	Clostridiaceae
354	29	48	16	60	58	278	320	206	122	170	281	197	Clostridiaceae
7	22	105	78	13	120	221	35	145	87	42	78	51	Lactobacillaceae
28	46	370	242	45	98	153	79	103	179	215	263	22	Lactobacillaceae
45	55	30	33	31	36	34	90	99	53	44	45	68	Prevotellaceae
0	97	6	1	5	0	10	115	3	103	2	127	214	Prevotellaceae
0	17	16	5	3	23	125	80	0	71	30	159	124	Prevotellaceae
235	363	5	4	0	5	871	0	162	207	266	36	331	Ruminococcacea
122	0	39	127	118	5	0	1	0	28	0	81	0	Ruminococcacea
22	0	1	52	3	34	115	59	33	48	63	58	84	Ruminococcacea
62	8	10	10	15	22	44	49	72	58	116	142	70	Turicibacteraceae
93	67	610	0	64	0	0	51	0	6	0	0	0	Verrucomicrobiaceae

Figure S3 Heat map showing the most abundant operational taxonomic units (OTUs, at least 500 total) in one lean subject, control, peach and plum groups. Colors represent differences in relative abundance within samples (red: higher; white: median; blue: lower).
