# Supplementary material for: Carbohydrate-Free Peach (Prunus persica) and Plum (Prunus domestica) Juice Affects Fecal Microbial Ecology in an Obese Animal Model
Source: PLoS One. 2014 Jul 9;9(7):e101723. doi: 10.1371/journal.pone.0101723 (PMC4090149; doi:10.1371/journal.pone.0101723)
Supplement: Table S1 — Total body weight at the end of the study (11 weeks of consumption of peach or plum juices), juice consumption and polyphenolics content in the control, peach, plum, and lean animal groups. (PDF) [file pone.0101723.s004.pdf]

Carbohydrate-free peach (*Prunus persica*) and plum (*Prunus domestica*) juice affects fecal microbial ecology in an obese animal model

Giuliana D. Noratto[1,a,b], Jose F. Garcia-Mazcorro[2,b], Melissa Markel[3], Hercia S. Martino[1], Yasushi Minamoto[3], Jörg M. Steiner[3], David Byrne[4], Jan S. Suchodolski[3] & Susanne U. Mertens-Talcott[1,5*]

1 Department of Nutrition and Food Science, Texas A&M University, College Station, Texas, United States of America

2 Facultad de Medicina Veterinaria y Zootecnia, Universidad Autónoma de Nuevo León, General Escobedo, Nuevo León, México

3 Gastrointestinal Laboratory, Texas A&M University, College Station, Texas, United States of America

4 Department of Horticultural Sciences, Texas A&M University, College Station, Texas, United States of America

5 Veterinary Physiology and Pharmacology, Texas A&M University, College Station, Texas, United States of America

[a] Current address: School of Food Science, Washington State University, USA.

[b] These authors contributed equally to this study.

*** Email**: SMTalcott@tamu.edu

Table S1 Total body weight at the end of the study (11 weeks of consumption of peach and plum juices), juice consumption and polyphenolics content in the control, peach, plum, and lean animal groups.

**Group**	**Body weight (g)**	**Juice consumption (mL/day)**	**Polyphenol content in juice (mg GAE/mL)**
Control	$644.4^{a} \pm 39.25$	50.6 ± 8.6	0
Peach	$611.1^{a} \pm 39.4$	47.5 ± 9.0	430 ± 6.3
Plum	$541.8^{b} \pm 43.6$	45.2 ± 12.6	1,270 ± 12.6
Lean	$385.7^{c} \pm 32.7$	46.6 ± 7.9	0

Values are mean (*n*=10) ± SD, different letters within the same column indicate statistically significant difference ($p<0.05$, ANOVA).
