# Supplementary material for: Carbohydrate-Free Peach (Prunus persica) and Plum (Prunus domestica) Juice Affects Fecal Microbial Ecology in an Obese Animal Model
Source: PLoS One. 2014 Jul 9;9(7):e101723. doi: 10.1371/journal.pone.0101723 (PMC4090149; doi:10.1371/journal.pone.0101723)
Supplement: Table S2 — Median (minimum-maximum) indices of bacterial diversity (Shannon Weaver and Chao1 3%) and richness (OTUs 3%) obtained from fecal samples of one lean subject, control, peach and plum groups. P values come from the non-parametric Kruskal-Wallis. (PDF) [file pone.0101723.s005.pdf]

Carbohydrate-free peach (*Prunus persica*) and plum (*Prunus domestica*) juice affects fecal microbial ecology in an obese animal model

Giuliana D. Noratto[1,a,b], Jose F. Garcia-Mazcorro[2,b], Melissa Markel[3], Hercia S. Martino[1], Yasushi Minamoto[3], Jörg M. Steiner[3], David Byrne[4], Jan S. Suchodolski[3] & Susanne U. Mertens-Talcott[1,5*]

1 Department of Nutrition and Food Science, Texas A&M University, College Station, Texas, United States of America

2 Facultad de Medicina Veterinaria y Zootecnia, Universidad Autónoma de Nuevo León, General Escobedo, Nuevo León, México

3 Gastrointestinal Laboratory, Texas A&M University, College Station, Texas, United States of America

4 Department of Horticultural Sciences, Texas A&M University, College Station, Texas, United States of America

5 Veterinary Physiology and Pharmacology, Texas A&M University, College Station, Texas, United States of America

[a] Current address: School of Food Science, Washington State University, USA.

[b] These authors contributed equally to this study.

*** Email**: SMTalcott@tamu.edu

Table S2 Median (minimum-maximum) indices of bacterial diversity (Shannon Weaver and Chao1 3%) and richness (OTUs 3%) obtained from fecal samples of one lean subject, control, peach and plum groups. *P* values come from the non-parametric Kruskal-Wallis.

	Lean*	Control (*n*=4)	Peach (*n*=4)	Plum (*n*=4)	P value
Chao1	699	512 (427-530)[a]	567 (561-598)[b]	487 (466-543)[a]	0.0244
Shannon	7.7	6.9 (6.5-7.4)	7.1 (6.7-7.5)	7.4 (6.9-7.4)	0.7939
OTUs	475	361 (326-410)	395 (392-410)	353 (334-406)	0.2628

These estimates are based on 2477-sequences subsamples. Different superscripts indicate statistical significance ($p<0.05$). *The estimates of the lean subject were not included in the statistical analysis.
