# Supplementary material for: Carbohydrate-Free Peach (Prunus persica) and Plum (Prunus domestica) Juice Affects Fecal Microbial Ecology in an Obese Animal Model
Source: PLoS One. 2014 Jul 9;9(7):e101723. doi: 10.1371/journal.pone.0101723 (PMC4090149; doi:10.1371/journal.pone.0101723)
Supplement: Table S3 — Median (minimum-maximum) relative proportions of pyrosequencing tags (percentage of sequences) for the control, peach, and plum groups. P values come from the non-parametric Kruskal Wallis test. (PDF) [file pone.0101723.s006.pdf]

Carbohydrate-free peach (*Prunus persica*) and plum (*Prunus domestica*) juice affects fecal microbial ecology in an obese animal model

Giuliana D. Noratto[1,a,b], Jose F. Garcia-Mazcorro[2,b], Melissa Markel[3], Hercia S. Martino[1], Yasushi Minamoto[3], Jörg M. Steiner[3], David Byrne[4], Jan S. Suchodolski[3] & Susanne U. Mertens-Talcott[1,5*]

1 Department of Nutrition and Food Science, Texas A&M University, College Station, Texas, United States of America

2 Facultad de Medicina Veterinaria y Zootecnia, Universidad Autónoma de Nuevo León, General Escobedo, Nuevo León, México

3 Gastrointestinal Laboratory, Texas A&M University, College Station, Texas, United States of America

4 Department of Horticultural Sciences, Texas A&M University, College Station, Texas, United States of America

5 Veterinary Physiology and Pharmacology, Texas A&M University, College Station, Texas, United States of America

[a] Current address: School of Food Science, Washington State University, USA.

[b] These authors contributed equally to this study.

*** Email**: SMTalcott@tamu.edu

Table S3 Median (minimum-maximum) relative proportions of pyrosequencing tags (percentage of sequences) for the control, peach, and plum animal groups. P values come from the non-parametric Kruskal Wallis test.

	Control (*n*=4)	Peach (*n*=4)	Plum (*n*=4)	p value
Phylum: Firmicutes	49(46-65)	50(38-55)	49(42-52)	0.8741
Clostridiaceae	15(11-25)	17(11-19)	16(12-18)	0.9033
Lactobacillaceae	8(3-14)	6(4-7)	7(2-9)	0.8741
Ruminococcaceae	9(5-10)	9(8-10)	8(6-11)	0.9569
Eubacteriaceae	5(4-16)	5(4-11)	6(4-7)	0.8741
Lachnospiraceae	4(3-7)	3(2-4)	3(2-4)	0.2341
Erysipelotrichaceae	1(0-2)	1(1-2)	2(2-4)	0.0359
Peptococcaceae	0(0-1)	0(0-1)	0(0-1)	0.8809
Bacillaceae	1(0-1)	1(0-2)	1(0-1)	0.3411
Paenibacillaceae	1(1-1)	1(1-1)	0(0-1)	0.2667
Oscillospiraceae	2(1-10)	4(2-6)	4(3-6)	0.4724
Catabacteriaceae	0(0-0)	0(0-0)	0(0-1)	0.2041
Turicibacter	0.3(0-0)[a]	0.9(1-1)[b]	2.1(1-3)[b]	0.0097
Phylum: Bacteroidetes	37(28-46)	44(42-57)	47(41-50)	0.1672
Prevotellaceae	4(1-11)	6(2-9)	8(3-10)	0.6831
Bacteroidaceae	17(9-20)	19(18-21)	16(12-21)	0.4908
Parabacteroides	0(0-1)	1(0-1)	1(0-1)	0.0775
Porphyromonadaceae	14(13-20)	17(13-33)	19(17-30)	0.2921
Rikenellaceae	1(1-1)	2(1-3)	1(1-2)	0.1692
Flexibacteraceae	0(0-0)	0(0-0)	0(0-0)	0.1108
Marinilabiaceae	0(0-0)	0(0-0)	0(0-0)	0.2973
Phylum: Proteobacteria	5(1-10)	4(2-8)	5(2-7)	0.7939
Desulfovibrionaceae	0(0-1)	0(0-1)	0(0-1)	0.6435
Sutterellaceae	4(0-8)	2(1-4)	4(0-6)	0.7774
Rhodospirillaceae	0(0-0)	0(0-2)	0(0-0)	0.6827
Alcaligenaceae	0(0-0)	0(0-0)	0(0-0)	0.1997
Anaplasmataceae	0(0-0)	0(0-0)	0(0-0)	0.1799
Pseudomonadaceae	0(0-0)	0(0-1)	0(0-0)	0.9834
Desulfuromonadaceae	0(0-0)	0(0-1)	0(0-0)	0.1923
Phylum: Tenericutes	0(0-1)	0(0-1)	0(0-0)	0.7895
Phylum: Actinobacteria	0(0-0)	0(0-0)	0(0-0)	0.6677
Coriobacteriaceae	0(0-0)	0(0-0)	0(0-0)	0.2559
Phylum: Verrucomicrobia	2(0-15)	0(0-1)	0(0-0)	0.0690
Verrucomicrobiaceae	2(0-15)	0(0-1)	0(0-1)	0.0906
Akkermansia	2(0-15)	0(0-1)	0(0-0)	0.0690
Phylum: Spirochaetes	0(0-1)	0(0-0)	0(0-1)	0.1738
Spirochaetaceae	0(0-1)	0(0-0)	0(0-1)	0.1687

This table only shows bacterial families and their respective phyla that were present in more than half the samples (i.e. at least 7 samples). A total of 33 bacterial families were found in very low abundance (less than 0.1% of all sequences) in only a few number of samples, and were therefore not included in this table. Additionally, this table shows three genera that reached (*Turicibacter*) or approached (*Parabacteroides* and *Akkermansia*) statistical significance. The rest of the genera did not approach statistical significance ($p>0.2$). Estimates with different superscripts are significantly different ($p <0.05$).
