# Supplementary material for: Carbohydrate-Free Peach (Prunus persica) and Plum (Prunus domestica) Juice Affects Fecal Microbial Ecology in an Obese Animal Model
Source: PLoS One. 2014 Jul 9;9(7):e101723. doi: 10.1371/journal.pone.0101723 (PMC4090149; doi:10.1371/journal.pone.0101723)
Supplement: Table S4 — Median (minimum-maximum) concentrations (μmol/g of wet feces) of short-chain fatty acids obtained from fecal samples of the control, peach, plum, and lean groups. P values come from the non-parametric Kruskal-Wallis. (PDF) [file pone.0101723.s007.pdf]

Carbohydrate-free peach (*Prunus persica*) and plum (*Prunus domestica*) juice affects fecal microbial ecology in an obese animal model

Giuliana D. Noratto[1,a,b], Jose F. Garcia-Mazcorro[2,b], Melissa Markel[3], Hercia S. Martino[1], Yasushi Minamoto[3], Jörg M. Steiner[3], David Byrne[4], Jan S. Suchodolski[3] & Susanne U. Mertens-Talcott[1,5*]

1 Department of Nutrition and Food Science, Texas A&M University, College Station, Texas, United States of America

2 Facultad de Medicina Veterinaria y Zootecnia, Universidad Autónoma de Nuevo León, General Escobedo, Nuevo León, México

3 Gastrointestinal Laboratory, Texas A&M University, College Station, Texas, United States of America

4 Department of Horticultural Sciences, Texas A&M University, College Station, Texas, United States of America

5 Veterinary Physiology and Pharmacology, Texas A&M University, College Station, Texas, United States of America

[a] Current address: School of Food Science, Washington State University, USA.

[b] These authors contributed equally to this study.

*** Email**: SMTalcott@tamu.edu

Table S4 Median (minimum-maximum) concentrations (µmol/g of wet feces) of short-chain fatty acids obtained from fecal samples of the control, peach, plum, and lean groups. P values come from the non-parametric Kruskal-Wallis.

	Control (*n*=5)	Peach (*n*=6)	Plum (*n*=8)	Lean (*n*= 5)	P value
Acetic acid	61.3[a] (46.5-92.3)	38.6[a,b] (27.0-68.2)	46.2[b,c] (24.8-59.9)	29.2[b,c] (25.3-45.1)	0.0439
Propionic acid	9.9[a] (6.4-10.9)	4.3[b] (0.0-7.8)	5.6[a,c] (2.5-10.3)	2.3[b] (0.0-7.1)	0.0184
Butyric acid	10.2 (5.1-14.0)	6.2 (1.9-11.5)	5.8 (2.1-8.5)	3.1 (1.5-10.6)	0.1864
Isobutyric acid	0.6 (0.0-0.8)	0.4 (0.1-0.9)	0.3 (0.1-0.8)	0.4 (0.2-1.7)	0.6564
Isovaleric acid	0.3 (0.2-0.4)	0.3 (0.2-0.5)	0.3 (0.2-0.5)	0.4 (0.1-1.4)	0.5858
Valeric acid	1.0 (0.4-1.3)	0.6 (0.2-0.9)	0.8 (0.3-1.1)	0.5 (0.2-1.6)	0.4575

Medians not sharing a common superscript are significantly different ($p<0.05$).
